# Low-dose lipopolysaccharide (LPS) inhibits aggressive and augments depressive behaviours in a chronic mild stress model in mice

**DOI:** 10.1186/s12974-016-0572-0

**Published:** 2016-05-16

**Authors:** Yvonne Couch, Alexander Trofimov, Natalyia Markova, Vladimir Nikolenko, Harry W. Steinbusch, Vladimir Chekhonin, Careen Schroeter, Klaus-Peter Lesch, Daniel C. Anthony, Tatyana Strekalova

**Affiliations:** Department of Pharmacology, Oxford University, Mansfield Road, OX1 3QT Oxford, UK; Division of Molecular Psychiatry, Laboratory of Translational Neuroscience, Department of Psychiatry, Psychosomatics and Psychotherapy, University of Würzburg, Würzburg, Germany; Department of Neuroscience, Maastricht University, Universiteitssingel 40, NL 6229 ER Maastricht, Netherlands; Institute of Physiologically Active Compounds, Moscow Region, Russia; Department of Anatomy, First Moscow Medical University, Moscow, Russia; Department of Basic and Applied Neurobiology, Serbsky Federal Medical Research Center for Psychiatry and Narcology, Moscow, Russia; Department of Preventive Medicine, Maastricht Medical Centre Annadal, Maastricht, Netherlands

**Keywords:** SERT, Chronic stress, LPS, Aggressive behaviour, 5-HT, Cytokines

## Abstract

**Background:**

Aggression, hyperactivity, impulsivity, helplessness and anhedonia are all signs of depressive-like disorders in humans and are often reported to be present in animal models of depression induced by stress or by inflammatory challenges. However, chronic mild stress (CMS) and clinically silent inflammation, during the recovery period after an infection, for example, are often coincident, but comparison of the behavioural and molecular changes that underpin CMS vs a mild inflammatory challenge and impact of the combined challenge is largely unexplored. Here, we examined whether stress-induced behavioural and molecular responses are analogous to lipopolysaccharide (LPS)-induced behavioural and molecular effects and whether their combination is adaptive or maladaptive.

**Methods:**

Changes in measures of hedonic sensitivity, helplessness, aggression, impulsivity and CNS and systemic cytokine and 5-HT-system-related gene expression were investigated in C57BL/6J male mice exposed to chronic stress alone, low-dose LPS alone or a combination of LPS and stress.

**Results:**

When combined with a low dose of LPS, chronic stress resulted in an enhanced depressive-like phenotype but significantly reduced manifestations of aggression and hyperactivity. At the molecular level, LPS was a strong inducer of TNFα, IL-1β and region-specific 5-HT_2A_ mRNA expression in the brain. There was also increased serum corticosterone as well as increased TNFα expression in the liver. Stress did not induce comparable levels of cytokine expression to an LPS challenge, but the combination of stress with LPS reduced the stress-induced changes in 5-HT genes and the LPS-induced elevated IL-1β levels.

**Conclusions:**

It is evident that when administered independently, both stress and LPS challenges induced distinct molecular and behavioural changes. However, at a time when LPS alone does not induce any overt behavioural changes per se, the combination with stress exacerbates depressive and inhibits aggressive behaviours.

**Electronic supplementary material:**

The online version of this article (doi:10.1186/s12974-016-0572-0) contains supplementary material, which is available to authorized users.

## Background

The association between depression and inflammation has been recognized for some time [1, 2]. Indeed, clinical trials have reported antidepressant treatment effects for anti-inflammatory agents such as non-steroidal anti-inflammatory drugs (NSAIDs), and pro-inflammatory cytokine inhibitors have also shown antidepressant treatment effects compared to placebo. Tumour necrosis factor alpha (TNFα) blockade, for example, improved depressive symptoms in patients with treatment-resistant depression, but only in patients with high baseline CRP levels [3], suggesting that the anti-inflammatory therapy targets processes independent of the etiological mechanisms underlying major depressive disorder (MDD). However, the additive nature of inflammation-induced depressive-like behaviours when combined with MDD highlights that inflammation is likely to be clinically relevant tractable target in many clinical forms of depression. However, while the impact of inflammatory challenges on the negative affect component of depression has been examined, the impact of inflammation on other accompanying behaviours has often been overlooked. Aberrant social behaviours, particularly aggression, as well as psychomotor agitation, often accompany depression and stress-related conditions in man and rodents [4–6]. Indeed, aggressive behaviour during major depression is associated with an enhanced risk of suicide [7]. Altered neuroimmune responses are also known to contribute to the neurobiology of aggression [8], and pro-inflammatory cytokine production, in particular, has been implicated in the mechanisms underpinning the stress response [5, 9] as well as aggressive behaviour [10–13].

Human and animal studies have linked aggression and impulsivity to the increased production of certain inflammatory mediators [11, 14]. In particular, aggressive traits in humans have been associated with increased serum TNFα [12], C-reactive protein [15] and other cytokines [16]. Indeed, patients in whom cytokines have been therapeutically administered often display signs of aggression [17, 18]. Furthermore, systemic expression of inflammatory mediators, such as increased systemic interleukin-1 beta (IL-1β) and interleukin-6 (IL-6), are associated with locomotor agitation during aging [11]. Conversely, mice selectively bred for high levels of aggression also display increased cytokine levels [19] and knockout of both TNFα-receptor-1 and TNFα-receptor-2 abrogates aggressive behaviours [20] suggesting that overall, cytokines and aggressive behaviours are linked. The finding that stress is associated with the induction of inflammation [21–23] could be interpreted in evolutionary terms, as a coherent mechanism to enhance survival. Stressors, such as predation, could potentially lead to injury and infection. Thus, pre-activation of the immune system would theoretically enhance survival and recovery [24].

In humans, parallels to the sickness behaviour observed upon systemic infection in rodents clearly exist. Interferon (IFN) therapy is known to induce transient signs of depression or malaise [25], and systemic inflammatory diseases are known to be accompanied by depressive-like signs [26, 27]. In rodents, CNS expression of pro-inflammatory cytokines IL-1β and TNFα contribute to anhedonia and behavioural measures of helplessness after chronic stress [28, 29]. Pro-inflammatory changes are associated with altered serotonergic function [30], over-expression of the 5-HT_2A_ receptor and over-expression of the serotonin transporter (SERT) [29], which together with other 5-HT-related elements underlie mechanisms of depressive symptoms and social dominancy [31]. Despite this, it remains unclear at what level and to what extent sickness behaviours and depression converge and how similar the underlying molecular profile is. For example, the impact of inflammation-induced depressive-like behaviour compared with chronic stress on measures of aggression and impulsivity or hyperactivity has been largely overlooked. Irrespective of whether the pathways leading to such aberrant behaviours are distinct, it is clear that a ‘double-hit’ of stress and infection impacts on the pathogenesis of depression [32, 33].

In the current study, we sought to determine the degree to which the behaviours associated with chronic mild stress (CMS) may be influenced by a mild, low-dose lipopolysaccharide (LPS) challenge that does not normally give rise to anything other than transient and subtle changes in behaviour that persist for no more than a couple of hours. MDD is a disease that is characterized by a recurrent episode of depression, but it is often unclear what factors might have precipitated relapse. Here, we were interested to discover how the single LPS challenge would impact on behaviour in a pre-stressed animal at a time when the effect of LPS had resolved. In this way, it is possible to evaluate the residual effects of acute inflammation on stress-induced behavioural changes. We examined behavioural parameters of aggression and impulsivity/hyperactivity, anhedonia and helplessness, as well as the expression of inflammatory and serotonergic markers of the periphery and specific brain areas, including the medial pre-frontal cortex and hippocampus as these sites are well recognized to play a crucial role in the stress response [34], and we have previously found that 5-HT_2A_ and SERT expression levels change in response to systemic inflammation [35] and chronic stress paradigms [29] in these regions.

A 10-day stress procedure was selected in the present study because it has been previously shown to induce a depressive-like syndrome in mice, which is accompanied by changes in CNS serotonergic and pro-inflammatory genes [29, 36]. Previous work in rats has shown that repeated LPS challenges, sufficient to induce sickness behaviour, when combined with chronic mild stress can induce additive increases in plasma corticosterone and TNFα in rats will enhance depressive-like behaviour [37]. In contrast to these findings in rats, our investigations have established, using a single low-dose LPS (0.1 mg/kg) in CMS mice and a broader set of behavioural tests, that there is no simple additive effect when inflammation and stress are combined but highlight selective independent effects on a number of stress-related behaviours and on the underlying molecular biology.

## Methods

### Animals

Studies were performed using 3.5-month-old male C57BL/6J mice; 3.5-month-old male CD1 mice were used as intruders for social stress, and 2–5-month-old Wistar rats were used for predator stress. All animals were supplied by the Gulbenkian Institute of Science, Oeiras, Portugal. C57BL/6J mice were housed individually for 14 days before the start of the experiments; CD1 mice and rats were housed in groups of five before the experiment and then individually thereafter. All animals were kept under a reversed 12-h light-dark cycle (lights on: 21:00 h) with food and water ad libitum, under controlled laboratory conditions (22 ± 1 °C, 55 % humidity). A minimum of six animals were used in all the behavioural experiments, and a minimum of five animals per group were used in the molecular biology experiments. All studies were carried out in accordance with the European Communities Council Directive for the care and use of laboratory animals upon approval by the ethics committee of Maastricht University for animal research (CPV, DEC-UM 2009-109) and permission 0421/000/000/2013 issued by the General Directory of Ethical Committee of the New University of Lisbon.

### Study outline

**Fig. 1:**
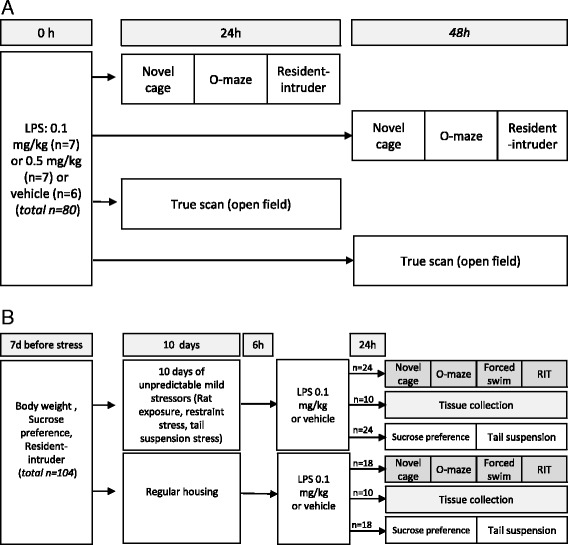
Schematic outline of the behavioural studies for **a** LPS-challenged stress-naïve mice and **b** LPS-challenged or vehicle-challenged stressed/non-stressed animals compared with control animals. The numbers in each group and total numbers are shown

The study design is outlined in the schematic in Fig. 1, and the animals were randomly assigned to test groups. The behavioural responses were studied in three separate cohorts. The first cohort was used to establish a subthreshold working dose of LPS that would not induce altered behaviour in the elevated O-maze and resident-intruder test in naïve mice, so that we would be able to explore the interaction of stress and LPS (Fig. 1a). A lack of immediate behavioural effects with 0.1 mg/kg LPS and a suppression of social and locomotor behaviour with 0.5 mg/kg LPS has been previously reported [38, 39]. The animals were subjected to either an acute LPS challenge, 0.5 or 0.1 mg/kg, or treated with vehicle alone and tested 24 h post-injection or, in order to explore the delayed behavioural response to LPS [40], 48 h post-injection, in separate subgroups. The second cohort (Fig. 1b) was subjected to chronic mild stress or no stress (minimal handling). Separate subgroups of mice were subjected to the novel cage, O-maze, forced swim test and a resident-intruder test or sucrose test and tail suspension or were killed for analysis of central and peripheral changes in gene expression and blood corticosterone. The stressed and non-stressed animals were then subsequently treated with either LPS at 0.1 mg/kg or vehicle 24 h prior to testing. The number of animals per group is indicated in the figure legends. A third cohort, duplicating the first, was employed to establish the effects of 0.1 and 0.5 mg/kg LPS on behaviour in the open field at 24 or 48 h post-injection using TruScan apparatus. Resting time and average speed were recorded.

### Acute LPS challenge

The animals were exposed to a single dose of LPS either 24 or 48 h prior to behavioural testing in the novel cage, O-maze or resident-intruder test. LPS (*E. coli* 0111:B4, Sigma-Aldrich) was made as a stock solution in sterile saline (0.9 %) and injected intraperitoneally (i.p.) at 0.1 or 0.5 mg/kg in a volume of 0.1 ml. Control animals received a single i.p. dose of saline (0.1 ml) to control for injection stress.

### Chronic mild stress

In the second (stressed) cohort, the animals underwent a previously validated 10-day chronic stress procedure [41]. The stress procedure consisted of rat exposure between the hours of 18:00 and 09:00 h (light phase of dark-light cycle) concomitant with a combination of restraint stress for 2 h and tail suspension for 40 min, applied in a semi-random manner with an inter-session interval of at least 4 h [29]. Briefly, during predation stress, mice were introduced to a transparent glass cylinder (15 cm high × ⌀ 8 cm) and placed into the rat cage for 15 h as described and validated previously [39, 40, 42]. For a restraint stress, mice were placed into a small container (50-ml Falcon tube) with space for breathing but no space for free movement, for 2 h, and for tail suspension, they were hung by their tails during the dark phase of the animals’ light cycle, as described previously [29]. Body weight, sucrose preference and previously defined social behaviour parameters were determined 1 week before the chronic stress procedure [38, 39, 41]. A further cohort of animals were killed, and tissue was collected for messenger RNA (mRNA) analysis.

### Behavioural testing

**Fig. 2:**
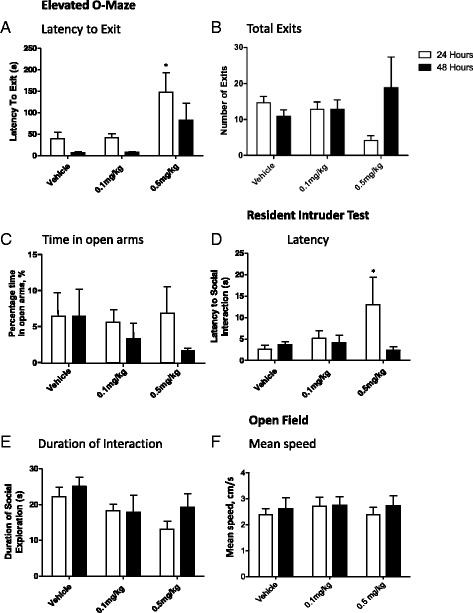
The effect of low doses of LPS on behavioural outcomes at 24 and 48 hours post-challenge innaïve mice. Animals were subjected to a single dose of LPS: 0.1 mg/kg or 0.5 mg/kg (n=7 in each group)or vehicle administration (*n*=6), and were tested 24 h or 48 h thereafter in the elevated O-maze for (**a**) latency to exit to open arm, (**b**) number of exits into open arms, and (**c**) the time spent in the open arms. Subsequently, mice were observed for (**d**) latency and (**e**) duration of social interaction in a resident-intruder test. Mean speed (**f**) was also recorder in the open field. Data are mean ± SEM; *p < 0.05 when compared to control animals

Behaviour was tested after 24 h because at this point, LPS-induced behavioural changes in stress-naïve mice had returned to baseline for the low-dose LPS challenge (Fig. 2). All behavioural testing was carried out during the dark phase of the animals’ light-dark cycle. Tests were recorded on film and analysis carried out post hoc and blinded, unless otherwise stated in the text.

### Elevated O-maze

The apparatus (Technosmart, Rome, Italy) consisted of a circular path (runway width 5.5 cm, diameter 46 cm) that was placed 45 cm above the floor. Two opposing arms were protected by walls (closed area, height 10 cm), and the illumination strength was 25 lx. The apparatus was placed on a dark surface in order to maintain control over lighting conditions during testing. Mice were placed in one of the closed-arm area of the apparatus. Behaviour was assessed using previously validated parameters during a 5-min observation period [36, 43]. The latency of the first exit to the ‘open’ compartments of the maze, the number of exits to the open arms and the percentage of time spent in the open arms were recorded [28].

### Resident-intruder test

The resident-intruder test procedure was adapted from previously described protocols [36, 44]. In this paradigm, the C57BL/6J mice were placed individually in an observation cage (30 × 60 × 30 cm) for 30 min to acclimatize. Thereafter, a previously group-housed male CD1 mouse was introduced as an intruder to the same cage and left with the resident mouse for 8 min. During the observation period, both resident mice were scored for the latency and duration of social interaction (nose-nose interactions) as well as the latency to attack, the number of attacks and the duration of crawl over behaviour. Crawl over time was recorded when the resident mouse positioned itself on top of the intruder mouse and often was usually associated with paw pressure on the head of the intruder [45]. During these periods, the intruder mouse showed little, if any, resistance to this mounting and displayed a submission by closing the eyes and not moving.

### Novel cage test

The novel cage test was performed to assess exploration in a new environment as described elsewhere [44, 46]. Mice were introduced into a standard plastic cage (21 × 21 × 15 cm) filled with fresh sawdust. The number of exploratory rears was counted under red light during a 5-min period.

### Open field TruScan

Mice were placed into TruScan activity boxes (26 × 26 × 39 cm; Coulbourn) for 10 min. The boxes were evenly illuminated with white light at 25 lx. Horizontal movements (speed) and resting time were scored automatically by red beam cells using TruScan software (Coulbourn), as described elsewhere [47]. Mean speed and total duration of resting behaviour, defined by a lack of crossing of more than three beams over 60 s, were evaluated.

### Sucrose preference

Mice were given 24 h of free choice between two bottles of either 1 % sucrose or standard drinking water, as described elsewhere [44]. The bottles were weighed before and after conducting the sucrose preference and consumption calculated accordingly. The beginning of the test started with the onset of the dark (active) phase of the animals’ cycle. To prevent the possible effects of side preference in drinking behaviour, the position of the bottles in the cage was switched at 12 h, halfway through testing. No prior food or water deprivation was applied before the test. Other conditions of the test were applied as described elsewhere [48]. Both baseline and post-stress paradigm sucrose preference tests utilized a 1 % sucrose solution. Percentage preference for sucrose was calculated at the end of the test using the following formula: Sucrose Preference = Volume_Sucrose solution_/(Volume_Sucrose solution_ + Volume_Water_) × 100.

### Tail suspension test

The protocol used in this study was adapted from a previously validated procedure [43, 47]. Mice were suspended by the tails to a rod 50 cm above the floor using adhesive tape. Animals were left on this apparatus for 6 min in a dark room. The apparatus was illuminated with a single spotlight (5 lx at animal height). The trials were recorded by a video camera positioned directly in front of the mice while the experimenter observed the session from a distance in a dark area of the experimental room. The total duration of this behaviour, a putative measure of ‘behavioural despair’, were scored using protocols that were previously validated with automated tools [43, 47]. In accordance with the commonly accepted criteria, immobility was defined as the absence of any movements of the animals’ head and body.

### Forced swim test

The protocol used for the Porsolt forced swim test was modified to prevent behavioural artefacts caused by stress-induced hyperlocomotion [41]. Mice were placed into a transparent pool (20 × 35 × 15 cm) lit with red light and filled with warm water (30 °C, to a depth of 9.5 cm) for 2 min. Floating behaviour, commonly interpreted as ‘behavioural despair’ in mice [49], was defined as the absence of directed movements of the animals’ head and body. Floating was measured by visual observation which was validated previously in comparison to automated scoring with specialized software [43, 47]. The latency to begin floating was scored as the time between introduction of the animal into the pool and the first moment of complete immobility of the entire body for a duration of >3 s. The total time spent floating was scored for the entire duration of the test using video footage.

### Tissue collection

Mice were terminally anaesthetized with an intraperitoneal injection of sodium pentobarbitone. The left ventricle was perfused in situ with 10 ml ice-cold saline; the brain and liver of each mouse were dissected. The pre-frontal cortex and striatum were collected by placing the brain, on its ventral side, on a metal plate. The olfactory bulbs were removed, and a 1-mm-thick coronal section of the most anterior cortical tissue was collected. The left and right cortical sections were further dissected to take the medial pre-frontal cortex while avoiding the motor cortex and anterior forceps of the corpus callosum. The left and right striatum was collected by generating a coronal section at bregma 0 and bregma +1. The cortex and corpus callosum were carefully removed and the left and right striatum collected. The hippocampus was removed by generating a coronal slice at bregma −1 and bregma −3. The overlying cortex was carefully removed, and the left and right hippocampus was removed. The dorsal raphe nucleus was collected from a 1-mm-thick section from bregma −4 to bregma −5 by collecting a diamond-shaped piece of tissue under the fourth ventricle. Small segments of liver tissue were isolated and stored at −80 °C.

### Quantitative RT-PCR (qPCR)

RNA extraction was performed as previously described from specifically microdissected snap-frozen brain regions and liver biopsies using the RNeasy Mini Kit (Qiagen, UK) [29]. The serotonergic genes 5-HT_2A_ and SERT were selected for analysis based on prior observation that their expression levels change in response to systemic inflammation [35] and chronic stress paradigms [29]. Primers were custom designed and synthesized, taking basic secondary structure into account during the design process (PrimerDesign Ltd., UK). All primers were validated against a standard complementary DNA (cDNA) biobank (PrimerDesign) to ensure adequate amplification and single melt-curve products. Five hundred nanograms of whole mRNA was converted to cDNA using random primers supplied with the High Capacity Reverse Transcription cDNA Kit (Applied Biosystems, UK), and final samples were diluted to 5 ng/μl. Standard curves were generated from a mixed cohort of cDNA, and analysis was performed using SYBR green (PrimerDesign Ltd., Southampton, UK) and a LightCycler 480 (Roche, UK). Cycle conditions were 8-min enzyme activation (95 °C), 15-s denaturation (95 °C), followed by 40 cycles of denaturation (15 s at 95 °C) and data collection (45 s at 60 °C). Total cDNA was used to enable normalization to expression of the housekeeping gene glyceraldehyde-3-phosphate (GAPDH) using the Pfaffl method [50]. Details of primers are listed in (Additional file 1: Table S1). Results are expressed as relative-fold compared to control animals.

### Corticosterone high-performance liquid chromatography (HPLC)

Blood was taken via cardiac puncture immediately before perfusion and stored in heparinized vials prior to centrifugation (10 k rpm, 10 min, 4 °C), and plasma was removed and immediately stored at −20 °C. Corticosterone was analysed using HPLC coupled with mass spectroscopy, based on the principles from Marwah et al. [51]. Briefly, plasma samples were diluted 1:1 with distilled water and applied to 2 ng of internal standard (5-pregnen-3b-ol-20-one-16a-carbonitrile). Diethyl ether was added to separate organic compounds into a water-free layer. Samples were vortexed and centrifuged (5 min, 1500×*g*) to fully separate solvent and aqueous layers. Solvent layers were removed and dried using a heated vacuum centrifuge. Organic residues were dissolved in 100 μl eluent A (see below) and applied to columns. Separations were carried out using a Waters 2695 separations module (Waters, Elstree, UK) with an ACE C18 3 μm, 100 × 2.1 mm column (Hichrom, Reading) maintained at 35 °C. The specific eluents were 2 mM acetic acid (A) and acetonitrile (B), with a linear gradient of 30–75 % of B over 8 min. The flow rate was 0.25 ml/min. The eluent was monitored using a Waters Micromass ZQ mass detector using positive electrospray ionization in single ion mode and Waters Empower 2 software. Mass spectrometry was performed under the following conditions: capillary voltage, 2.7 kV; source temperature, 125 °C; desolvation temperature, 475 °C; desolvation gas flow, 575 l/h; and cone gas flow, 80 l/h. Corticosterone was monitored at *m*/*z* 347.1 (*M* + *H*), cone voltage 20 V. The internal standard CA4 was monitored at *m*/*z* 302.1, cone voltage 35 V.

### Statistics

Data were analysed using GraphPad Prism version 6.0 for Windows (San Diego, CA) and InVivoStat software. Two-way ANOVA and RM-ANOVA were used followed by post hoc tests as appropriate (Bonferroni) and as indicated in the text. The level of confidence was set at 95 % (*p* < 0.05), and data are shown as mean ± SEM.

## Results

### A low dose of LPS of 0.1 mg/kg has no significant behavioural effects in naïve mice

Naive mice were challenged with either 0.1 or 0.5 mg/kg LPS to determine the behavioural effects of each dose 24 or 48 h thereafter [35]. In the elevated O-maze, there was a main effect of LPS dose, but not time post-challenge, on the overall latency to exit to the open arms (Fig. 2a; two-way ANOVA dose *p* < 0.01 *F*_2,34_ = 7.89; time post-challenge *p* = 0.06 *F*_1,34_ = 0.55; dose:time post-challenge *p* = 0.78 *F*_2,34_ = 0.25). Post hoc analysis demonstrates that at 24 h post-challenge, 0.5 mg/kg LPS animals have a significantly increased latency to exit to the open arms compared to controls (Fig. 2a; Bonferroni *p* < 0.05). The total number of exits from the closed area of the O-maze was not affected by either LPS dose or time (Fig. 2b; two-way ANOVA dose *p* = 0.93 *F*_2,34_ = 0.07; time post-challenge *p* = 0.28 *F*_1,34_ = 1.18; dose:time post-challenge *p* = 0.07 *F*_2,34_ = 2.90). There was a non-significant tendency for the higher dose of LPS to affect the total number of exits at 24 h post-challenge; this was not significant (Fig. 2b; *p* = 0.07). The proportion of time spent in the open arms of the elevated O-maze was also unaffected by treatment (Fig. 2c; two-way ANOVA dose *p* = 0.60 *F*_2,34_ = 0.5.14; time post-challenge *p* = 0.25 *F*_1,34_ = 1.343; dose:time post-challenge *p* = 0.63 *F*_2,34_ = 0.455).

In the resident-intruder test, there was no overall effect of dose or time on social interaction (Fig. 2d; two-way ANOVA dose *p* = 0.31 *F*_2,34_ = 1.18; time post-challenge *p* = 0.16 *F*_1,34_ = 2.06; dose:time post-challenge *p* = 0.13 *F*_2,34_ = 2.14), but post hoc testing showed a significantly increased latency of social interaction in animals receiving 0.5 mg/kg and tested at 24 h when compared to vehicle-treated controls (Fig. 2d; Bonferroni *p* < 0.05). The total time spent interacting with the intruder was also not affected by LPS at either 24 or 48 h (Fig. 2e; two-way ANOVA dose *p* = 0.07 *F*_2,34_ = 2.89; time post-challenge *p* = 0.26 *F*_1,34_ = 1.27; dose:time post-challenge *p* = 0.57 *F*_2,34_ = 0.57). Using open field and novel cage tests, we observed no effect of either dose on locomotor activity on mean speed (Fig. 2f; two-way ANOVA dose *p* = 0.77 *F*_2,34_ = 0.257; time post-challenge *p* = 0.47 *F*_1,34_ = 0.53; dose:time post-challenge *p* = 0.90 *F*_2,34_ = 0.10) or on resting time or the number of rears (Additional file 1: Figure S1).

### Stress-induced depressive-like behaviours tend to be exacerbated by systemic inflammation

**Fig. 3:**
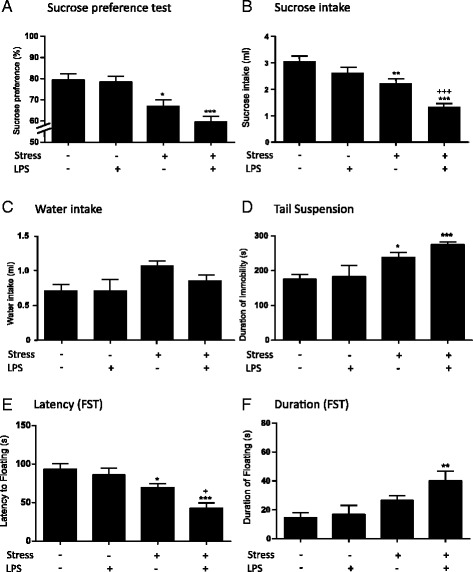
The effect of low-dose LPS on depressive-like behaviours in stressed mice. Naïve and stressed animals were subjected to either a single dose of LPS (0.1 mg/kg) or vehicle injection and tested 24 h thereafter in a two-bottle sucrose preference test investigating **a** overall preference for sucrose, **b** total sucrose consumption, **c** water intake in a sucrose test, **d** the period of immobility in the tail suspension test, and in the forced swim test for **e** latency to floating and **f** total time spent floating. All animals showed >65 % preference for sucrose at baseline and similar sucrose preference prior to bolus injection of LPS or vehicle (Additional file 1: Figure S2). Data are mean ± SEM; *p < 0.05, **p < 0.01 and ***p < 0.001 when compared to controls; +*p* < 0.05 and +++*p* < 0.001 compared to stressed animals

Since the lower dose of LPS did not affect the behaviour of naïve mice at 24 h, it was used for the chronic stress study. We first assessed body weight (experimental groups were balanced at baseline) and showed that stress reduced body weight as expected (Additional file 1: Figure S2). Low-dose LPS (0.1 mg/kg) given 24 h prior to testing does not significantly alter parameters of sucrose preference test (Additional file 1: Figure S2) [41]. However, it was hypothesized that if stress increases pro-inflammatory cytokines, stimulation of the system with an inflammatory challenge may significantly alter this behaviour. All animals showed a preference of >65 % for a 1 % sucrose solution prior to testing and a consistent sucrose and water intake (Additional file 1: Figure S2 C–E). Control animals, and animals injected 24 h prior to testing with 0.1 mg/kg LPS, maintained a sucrose preference of >65 % and were not significantly different from each other (Fig. 3a). After 10 days of chronic stress, there was a significant main effect of stress on sucrose consumption but not of LPS, and there was no interaction between stress and LPS (Fig. 3a; two-way ANOVA; stress *p* < 0.001 *F*_1,54_ = 16.62; LPS *p* = 0.28 *F*_1,54_ = 1.182; stress:LPS *p* = 0.41 *F*_1,54_ = 0.689). Consistent with the main effects, post hoc tests showed that after 10 days of chronic stress and a single i.p. dose of saline, animals displayed a significant decrease (<65 %) preference for a sucrose solution (Fig. 3a; Bonferroni post hoc *p* < 0.05). Post hoc analysis revealed that animals undergoing 10 days of chronic stress combined with a single i.p. dose of LPS (0.1 mg/kg) 24 h prior to testing also showed a decrease in sucrose preference compared to controls (*p* < 0.001). While there appears to be a decrease in sucrose preference for stressed animals receiving LPS compared to those without LPS, this difference is not significant (*p* = 0.192). Since sample sizes are unequal across groups, post hoc tests should be considered with caution. Total sucrose intake somewhat reflects this, here showing a main effect of both stress and LPS but no interaction (Fig. 3b; two-way ANOVA; stress *p* < 0.001 *F*_1,54_ = 25.36; LPS *p* < 0.01 *F*_1,54_ = 10.27; stress:LPS *p* = 0.28 *F*_1,54_ = 1.15). Post hoc testing revealed a significant decrease in sucrose consumption in stressed animals when compared to non-stressed controls (Fig. 3b; Bonferroni post hoc *p* < 0.01). Post hoc testing also reveals a decreased sucrose intake in stressed mice treated with LPS when compared to those treated with vehicle, suggesting a higher degree of anhedonia in these animals (Fig. 3b; Bonferroni post hoc *p* < 0.0001). Finally, stressed mice show some degree of hyperdipsia, with water consumption being affected by stress, but not by any other factors (Fig. 3c; two-way ANOVA; stress *p* < 0.05 *F*_1,54_ = 5.38; LPS *p* = 0.31 *F*_1,54_ = 1.04; stress:LPS *p* = 0.35 *F*_1,54_ = 0.85).

Tail suspension is used to measure helpless behaviour, which is associated with a depressive-like state in mice [52, 53]. Analysis showed a significant effect of stress on the total time spent immobile in the test (Fig. 3d; two-way ANOVA; stress *p* < 0.001 *F*_1,40_ = 24.89; LPS *p* = 0.16 *F*_1,40_ = 1.97; stress:LPS *p* = 0.35 *F*_1,40_ = 0.89) but no other main effects and no interactions. Post hoc testing showed that all stressed animals, irrespective of treatment, were immobile for significantly longer periods than controls (Fig. 3d; Bonferroni post hoc; stress *p* < 0.05; stress and LPS *p* < 0.0001).

In the forced swim test, another test for helpless behaviour, control and LPS-alone animals showed similar values in both the latency to float and total time spent floating. Analysis showed that both stress and LPS had a main effect on the latency to floating behaviour but that there was no interaction between factors and therefore, all results should be considered with caution (Fig. 3e; two-way ANOVA; stress *p* < 0.001 *F*_1,43_ = 21.46; LPS *p* < 0.05 *F*_1,43_ = 5.495; stress:LPS *p* = 0.19 *F*_1,43_ = 1.76). In post hoc tests, chronic stress significantly decreased the latency to float compared to controls (Fig. 3e; Bonferroni post hoc; *p* < 0.05), as did chronic stress combined with LPS (*p* < 0.001). Using multiple pairwise comparisons (Bonferroni post hoc), LPS combined with stress is significantly different from stress alone (*p* < 0.05); however, as there is no interaction between these factors, this result should be interpreted with caution.

There was a main effect of stress, not LPS, on the total duration of floating behaviour, and there was no interaction between factors (two-way ANOVA; stress *F*_1,43_ = 9.654, *p* < 0.01; LPS *F*_1,43_ = 1.922, *p* = 0.17; stress: LPS *F*_1,43_ = 0.99, *p* = 0.32; Fig. 3f). In post hoc tests, the combination of chronic stress and LPS significantly increased the total time spent floating compared to the control group in the forced swim test (Fig. 3f; Bonferroni post hoc; *p* < 0.05). While this suggests that LPS combined with stress significantly affects floating behaviour in the forced swim test, the lack of interaction makes these results difficult to interpret.

### Inflammation decreases aggression and impulsivity in stressed animals

**Fig. 4:**
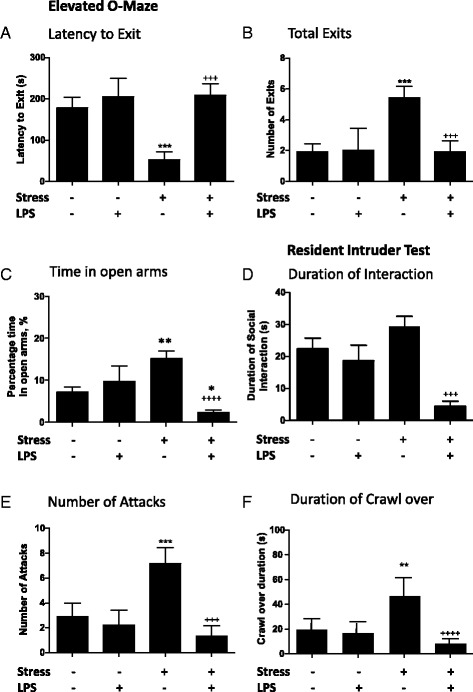
The effect of low-dose LPS on anxiety and aggression-like behaviours in stressed mice. Naïve and stressed animals were challenged with a single dose of LPS (0.1 mg/kg) or vehicle (saline) and tested 24 h thereafter in the elevated O-maze for the **a** latency to exit to the open arms and **b** number of exits to the open arms; in the resident-intruder paradigm for **c** duration of social interaction and **d** latency to attack conspecific, **e** total number of attacks and **f** duration of crawl over behaviour. Data are mean ± SEM; *p < 0.05, **p < 0.01 and ***p < 0.001 compared to control animals; +++*p* < 0.001 and ++++*p* < 0.0001 compared to stressed animals

In the O-maze, stress and LPS significantly affected the latency to exit into the open arms independently and through interaction (Fig. 4a; two-way ANOVA; stress *F*_1,39_ = 4.41, *p* < 0.05; LPS *F*_1,39_ = 9.84, *p* < 0.01; stress: LPS *F*_1,39_ = 4.87, *p* < 0.05). In stressed animals, LPS reversed the stress-induced decrease in the latency to exit to the open arms, ameliorating this parameter which is an assumed sign of impulsivity (Fig. 4a; Bonferroni post hoc; *p* < 0.001). Similarly, the total number of exits to the open arms of the maze was significantly affected by stress and LPS independently and in terms of interaction (Fig. 4b; two-way ANOVA; stress *F*_1,39_ = 4.55, *p* < 0.05; LPS *F*_1,39_ = 4.58, *p* < 0.05; stress: LPS *F*_1,39_ = 5.01, *p* < 0.05). Post hoc testing also demonstrated that the presence of LPS significantly diminished the number of exits to the open arms of the O-maze in stressed animals, thus abolishing the impulsivity/hyperlocomotion in these mice (Fig. 4b; Bonferroni post hoc; *p* < 0.01).

The resident-intruder test can be used to assess both social and aggressive behaviours [54]. Resident-intruder testing was performed on all animals before undertaking the chronic stress and/or dosing procedure, and all groups were shown to be balanced at baseline (Additional file 1: Figure S3). We found that the duration of social exploration was significantly decreased by LPS and there was also an interaction between stress and LPS (Fig. 4d; two-way ANOVA; stress *F*_1,35_ = 1.17, *p* = 0.28; LPS *F*_1,35_ = 18.81, *p* < 0.0001; stress: LPS *F*_1,35_ = 10.15, *p* < 0.01). Post hoc testing found that stressed animals challenged with LPS interacted with their intruders for significantly less time than those not challenged with LPS (Fig. 4d; Bonferroni post hoc; *p* < 0.0001).

When aggressive behaviour was examined, we found that the 10 days of chronic stress increased crawl over behaviour and the number of attacks and this was significantly inhibited by LPS treatment (Fig. 4). LPS treatment affected the number of the total number of attacks compared to control animals in an independent fashion, and analysis revealed a further interaction with stress (Fig. 4e; two-way ANOVA; stress *F*_1,35_ = 1.89, *p* = 0.17; LPS *F*_1,35_ = 7.16, *p* < 0.01; stress: LPS *F*_1,35_ = 4.39, *p* < 0.05). LPS significantly reduced the stress-induced rise in the number of attacks analysed with post hoc testing (Fig. 4e; Bonferroni post hoc; *p* < 0.001). Crawl over behaviour, a measure of a dominant-like interaction [45], was found to be increased in the animals exposed to stress, and this was once more significantly reduced in the stressed animals that were challenged with LPS (Fig. 4f; RM-ANOVA; stress/LPS treatment *F*_3,35_ = 3.59, *p* < 0.05; before/after *F*_1,35_ = 2.85, *p* = 0.1; stress/LPS: before/after *F*_3,35_ = 6.78, *p* < 0.01). Stressed animals showed an increased amount of crawl over behaviour when compared to controls (Fig. 4f; Bonferroni post hoc *p* < 0.01). Furthermore, stressed animals treated with LPS showed significantly less crawl over behaviour when compared to animals that had undergone stress alone (Fig. 4f; Bonferroni post hoc *p* < 0.0001).

Behaviour in a novel cage was also examined in all animals. Those animals undergoing 10 days of chronic stress followed by either an LPS challenge or a vehicle challenge showed no significant change in rearing behaviour in this test (two-way ANOVA; stress *F*_1,32_ = 1.29, *p* = 0.26; LPS *F*_1,32_ = 0.01, *p* = 0.9; stress: LPS *F*_1,32_ = 0.17, *p* = 0.67). This suggests that the changes observed in behavioural tests for aggression or social interaction above were unlikely to be a result of confounding alterations in general locomotor activity (Additional file 1: Figure S2).

### Inflammation and stress cumulatively increase hepatic IL-1β, but not corticosterone

Systemic inflammation has been shown to increase circulating cytokines, and stress is known to decrease pro-inflammatory cytokine expression via glucocorticoid induction [55]. As mentioned above, the levels of pro-inflammatory cytokines present 24 h after injection of 0.1 mg/kg endotoxin should be relatively low [56].

**Fig. 5:**
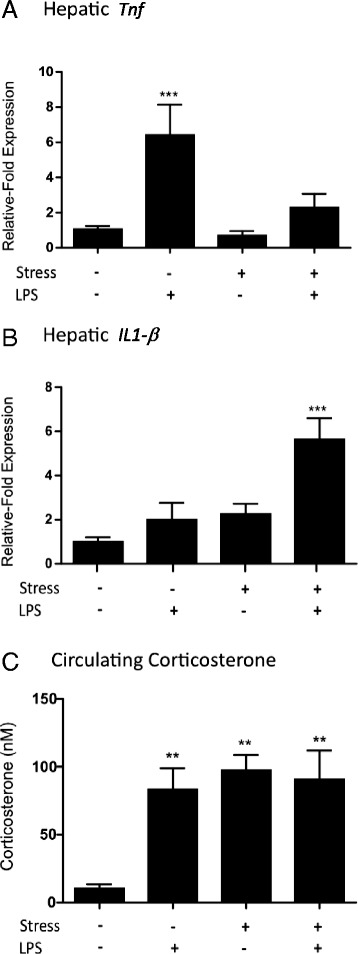
Cytokine mRNA in the liver and blood corticosterone levels in control, stressed and LPS-treated animals. mRNA levels of **a** TNFα and **b** IL-1β were measured by qPCR in the liver of animals after either 10 days of chronic stress, an acute LPS challenge (0.1 mg/kg) or a combination of both. Corticosterone levels in blood (**c**) were measured by HPLC. qPCR data are expressed as relative-fold expression normalized to GAPDH and naïve mice. *Bars* are mean ± SEM, (*n* = 5 in each group), **p < 0.01 and ***p < 0.001 compared to control animals

In this experiment, both LPS and stress had a significant effect on TNFα gene expression; furthermore, there was a significant interaction between the factors (Fig. 5a; two-way ANOVA; stress *p* < 0.01 *F*_1,18_ = 9.259; LPS *p* < 0.001 *F*_1,18_ = 22.07; stress:LPS *p* < 0.05 *F*_1,18_ = 6.472). At 24 h after LPS injection in non-stressed mice, the fivefold increase in hepatic *Tnf* compared to vehicle-treated controls was statistically significant (Fig. 5a; Bonferroni post hoc; *p* < 0.001). Chronic stress and LPS, combined, appeared to the levels of TNFα mRNA compared to vehicle controls, but this change was not significant (Fig. 5a).

IL-1β mRNA expression was affected by stress and LPS, but there was no significant interaction between the two factors (Fig. 5b; two-way ANOVA; stress *p* < 0.001 *F*_1,18_ = 15.56; LPS *p* < 0.01 *F*_1,18_ = 12.61; stress:LPS *p* = 0.07 *F*_1,18_ = 3.711). IL-1β mRNA expression was slightly higher in animals treated with stress and LPS alone but in neither case are they significantly different from non-stressed, vehicle-treated controls (Fig. 5b). The combination of 10 days of chronic stress and a low-dose LPS challenge resulted in a significant sixfold increase in hepatic IL-1β mRNA expression (Fig. 5b; Bonferroni post hoc; *p* < 0.001).

Control animals had an average of 10-nM baseline corticosterone (Fig. 5c). Both stress and LPS had a significant effect on corticosterone levels, and there was a significant interaction between these factors (Fig. 5c; two-way ANOVA; stress *p* < 0.05 *F*_1,25_ = 4.605; LPS *p* < 0.01 *F*_1,25_ = 9.355; stress:LPS *p* < 0.05 *F*_1,25_ = 6.659). More specifically, analysis showed that administration of 0.1 mg/kg LPS significantly increased circulating corticosterone when compared to controls, to an average of 90 nM (Fig. 5c; Bonferroni post hoc; *p* < 0.01). Following 10 days of stress and 10 days of stress in combination with an LPS challenge, elevated circulating corticosterone levels (100 nM) were also found and were significantly higher than controls (Fig. 5c; Bonferroni post hoc; stress alone *p* < 0.01, stress and LPS *p* < 0.01). At no point were stressed or LPS-treated animals different from each other, and stress combined with LPS did not result in an additive increase in corticosterone concentration.

### Low-dose LPS-induced inflammation does not exacerbate chronic stress-induced changes in 5-HT_2A_ and SERT expression or CNS cytokine expression

Previous work from our laboratory has demonstrated that both LPS and chronic stress are independently capable of changing the expression of the 5-HT_2A_ receptor and SERT mRNA expression [29, 35]. The data above demonstrate that LPS is capable of exacerbating certain behaviours induced by the chronic stress. Therefore, it is important to determine whether receptor expression was also cumulatively increased or whether, like corticosterone, low-level inflammation in stressed animals did not affect receptor expression. The addition of both stress and LPS into the model requires a more complex analysis with stress, LPS and brain regions as repeated factors. The general linear model applied to the earlier data remains with unstructured co-variance but with the added capacity of determining whether stress and LPS interact with each other. The number of possible interactions makes reporting this data rather excessive; therefore, only significant values are reported below.

**Fig. 6:**
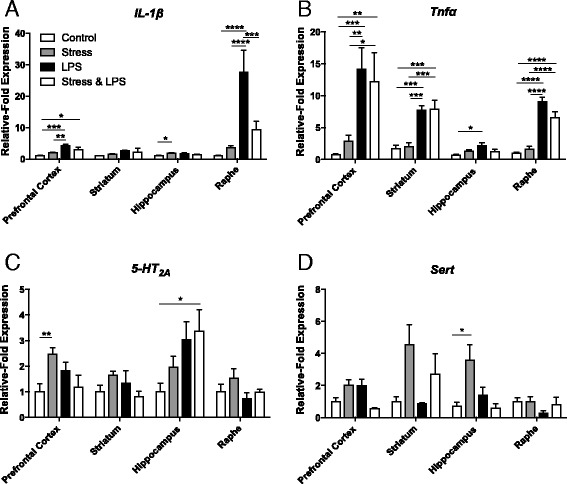
IL-1β, TNFα, 5-HT_2A_ receptor and SERT mRNA expression in the brain structures of animals challenged with chronic stress, LPS or a combination of both. mRNA levels of **a** IL-1β, **b** TNFα, **c** 5-HT_2A_ and **d** SERT were measured by qPCR in the pre-frontal cortex, striatum, hippocampus and raphe of animals after either 10 days of chronic stress, an acute LPS challenge (0.1 mg/kg) or a combination of both. Values are expressed as relative-fold expression normalized to housekeeping gene GAPDH and to control values within each region. Data are mean ± SEM; *n* = 5 in each group; *p < 0.05, **p < 0.01, ***p < 0.001 and ****p < 0.0001 compared to control animals

IL-1β mRNA levels were significantly affected by both stressors, either stress or LPS alone or combined and by brain region (Fig. 6a; RM-ANOVA brain region *p* < 0.001 *F*_4,48_ = 16.91; stress:LPS:brain region *p* < 0.001 *F*_4,48_ = 13.69). These factors also showed a significant interaction, suggesting that stress/LPS had a differential effect on IL-1β mRNA levels in different brain regions (Fig. 6a; brain region:stressor *p* < 0.001 *F*_4,48_ = 8.58). Post hoc testing revealed significant effects of LPS alone, and stress combined with LPS, in the dorsal raphe nucleus (Fig. 6a; Bonferroni post hoc; *p* < 0.001 stress vs LPS; *p* < 0.05 control vs stress and LPS), and these differences continued in the raphe when comparing animals that were only stressed for 10 days to animals that were stressed but also challenged with LPS (Fig. 6a; Bonferroni post hoc *p* < 0.001). Other brain regions only showed minor increases in IL-1β receptor mRNA expression after either stress or LPS, and these did not reach significance (Fig. 6a). However, it should be cautioned that large changes in any individual brain region, such as the raphe, are likely to mask smaller changes in other brain regions.

In the brain, TNFα mRNA expression was affected in a similar manner to IL-1β mRNA expression, with significant main effects of both brain region and stressor and a significant interaction (Fig. 6b; RM-ANOVA brain region *p* < 0.05 *F*_4,48_ = 15.64; stressor *p* < 0.01 *F*_4,48_ = 7.72; stress:LPS:brain region *p* < 0.05 *F*_4,48_ = 2.89). Post hoc testing suggests that stress alone does not exacerbate TNFα mRNA expression but as with the IL-1β results, larger changes in other regions may mask specific effects. LPS administration induced a significant increase in TNFα mRNA expression in all regions, with the exception of the hippocampus (Fig. 6b; Bonferroni post hoc; pre-frontal cortex *p* < 0.0001; striatum *p* < 0.01; raphe *p* < 0.0001). Stress combined with an inflammatory challenge results in a significant increase in TNFα expression in similar regions compared to control (Fig. 6b; Bonferroni post hoc; pre-frontal cortex *p* < 0.001; striatum *p* < 0.01; raphe *p* < 0.05). Finally, there were significant differences between stress-alone animals and animals stressed and challenged with LPS but only in the pre-frontal cortex (Fig. 6b; Bonferroni post hoc *p* < 0.01). However, there was no overt synergy between stress with LPS and LPS alone.

Analysis shows that there was only a significant main effect of brain region on 5-HT_2A_ mRNA expression, as well as a significant interaction between brain region, stress and LPS challenge (Fig. 6c; RM-ANOVA brain region *p* < 0.001 *F*_4,48_ = 16.20; stress:LPS:brain region *p* < 0.01 *F*_4,48_ = 4.96). Post hoc analysis shows 5-HT_2A_ receptor mRNA expression appeared to increase after a single LPS injection in the pre-frontal cortex, striatum and hippocampus, compared to controls, but was only significantly different in the hippocampus (Fig. 6c; Bonferroni post hoc; *p* < 0.01). There was no difference, significant or otherwise, in 5-HT_2A_ mRNA levels in the raphe compared to controls (Fig. 6c). In a similar manner, after 10 days of chronic stress, 5-HT_2A_ mRNA appeared to be elevated in the pre-frontal cortex as well as the hippocampus but again, only reached significance in the latter when compared to control animals (Fig. 6c; Bonferroni post hoc; *p* < 0.01). Chronic stress did not change receptor expression in either the striatum or the raphe. In the CNS of animals challenged with 10 days of chronic stress and LPS, 5-HT_2A_ receptor mRNA expression was not different from controls in any region except the hippocampus, where it showed an increase of a similar magnitude to stress and LPS alone (Fig. 6c; Bonferroni post hoc; *p* < 0.05).

SERT mRNA expression showed the same main effects as for the 5-HT_2A_ receptor, but significant interactions were noted. Specifically, there was a main effect of brain region and interactions between brain region and stress, brain region and LPS challenge and all three factors (Fig. 6d; RM-ANOVA; brain region *p* < 0.001 *F*_4,48_ = 22.23; stress:brain region *p* < 0.001 *F*_4,48_ = 15.46; LPS:brain region *p* < 0.001 *F*_4,48_ = 6.42; stress:LPS:brain region *p* < 0.01 *F*_4,48_ = 12.32). Further analysis showed that SERT expression in the pre-frontal cortex after a single LPS challenge appeared to be higher than controls but did not reach significance (Fig. 6d; Bonferroni post hoc pre-frontal cortex *p* = 0.081). No other brain regions studied showed any change in SERT mRNA compared to controls after a single dose of LPS. Ten days of chronic stress did not change SERT expression in the pre-frontal cortex or the raphe compared to controls but led to significantly higher expression in the striatum and hippocampus (Fig. 6d; Bonferroni post hoc; striatum *p* < 0.001, hippocampus *p* < 0.01). Compared to stress alone, the combination of stress and LPS did give rise to any significant increase SERT mRNA expression in any region studied (Fig. 6d).

## Discussion

The studies reported here show that at a time when the effects of an intraperitoneal injection of LPS are no longer detectable in naïve animals, the combination of LPS with CMS increases depressive-like behaviours and inhibits the aggression and impulsivity induced by CMS. The aggressive and impulsive behaviours were accompanied by SERT induction in the hippocampus, which was ameliorated by the LPS treatment. The double-hit combination had no effect on LPS-induced TNFα expression but did suppress LPS-induced IL-1β mRNA expression. Overall, SERT upregulation, rather than 5-HT_2A_ or the pro-inflammatory cytokines, appears to correlate with the stress-induced aggressive and impulsive behaviours. A similar independent increase in SERT was previously reported in stressed animals that become anhedonic [29]. Here, hepatic TNFα and IL-1β mRNA levels differed between stressed mice injected with LPS compared to LPS alone in a surprising manner revealing a dissociation between the regulation of TNFα and IL-1β mRNA expression. Moreover, these changes in hepatic cytokine expression appeared to be independent of corticosterone induction. These results are discussed in more detail below.

Using a low-dose LPS challenge after stress in both the sucrose preference test and the forced swim test, we showed that the downstream sequelae of a peripheral inflammatory response appeared to exacerbate the anhedonia and helplessness induced by stress. Indeed, there was significant synergy for a reduction in sucrose intake. Non-stressed mice exhibit polydipsia, and this in known to be reduced by stress [57] and is further reduced by the LPS challenge, indicating that low levels of systemic inflammation that may not generate overt clinical signs per se can synergize with a stress-induced depressive illness and provoke a worsening phenotype. Thus, the diagnosis and treatment of low-grade inflammatory disease in patients may reduce some select depressive signs by mechanisms that are independent of those that are associated with major depression and those targeted by traditional antidepressants. Others have shown that the combination of endotoxin with stress in mice can result in increased mortality [58] but such severe experiments (40 mg/kg LPS compared to 0.1 mg/kg in our studies) did not set out to explore the subtle relationship between low-level infection and stress. In rats, lower levels of endotoxin were previously used to discover how inescapable shock-induced stress would be altered [59, 60]. In these experiments, the febrile response associated with inescapable shock and LPS was increased and this was associated with enhanced pro-inflammatory cytokine responses. As in our experiments, Johnson et al. [60] found the relationship between cytokine expression and the double-hit of stress and inflammation was not a straightforward relationship; enhanced pro-inflammatory cytokine responses where not necessary to observe enhanced HPA or fever responses after LPS and inescapable tailshock.

Work studying the immune response after a stressful event has suggested that stress ‘primes’ the inflammatory response for an immune challenge, making it more sensitive [15]. The depressive-like behaviours associated with an LPS challenge have also been shown to be ameliorated by imipramine and fluoxetine given prior to LPS administration [61], and our results suggest that while antidepressants might target the post-infection component of the combination, anti-inflammatory therapy might also be beneficial. Indeed, celecoxib administered as an adjunctive non-steroidal anti-inflammatory drug (NSAID) appears to produce a positive therapeutic outcome in the treatment of depression [62].

In this study, although chronically stressed mice exhibit anhedonia, they also display increased rates of aggressive behaviour in the resident-intruder test where attacking and crawl over behaviours were markedly increased. Crawl overs have been investigated in rats and form part of juvenile play fighting. However, such behaviour has also been observed in aggressive encounters. In rats, crawl overs occur when the rats are unfamiliar with one another and seem to be important in establishing dominance [45]. Such stress-induced changes in attack frequency have been previously described using the same CMS regime as employed here [36]. A paradoxical ‘anxiolytic-like profile’, manifest as increased impulsivity in the elevated O-maze, was also observed in response to stress, in line with previously reported findings [63]. In contrast, stressed mice subjected to a low-level LPS challenge displayed a reduction in aggressive behaviour in the resident-intruder test and no signs of impulsivity/hyperlocomotion in the elevated O-maze. In studies of aggression and impulsivity, the combination of stress and low-level inflammation therefore appears to counteract, rather than exacerbate, the negative effects of stress on behaviour.

Changes in measures of aggressiveness and impulsivity/hyperactivity were accompanied by differential expression of SERT in the brain. In the hippocampus, mRNA levels of SERT were increased in chronically stressed mice. In stressed mice challenged with LPS, expression levels of SERT in the hippocampus did not change, but they did tend towards a decrease in the pre-frontal cortex. Chronically stressed mice without exposure to LPS displayed a non-significant increase in SERT expression in the pre-frontal cortex. These data are in accordance with our previous observations [30]. Elevated SERT expression was previously reported in mice displaying aggressive behaviour induced by repeated social confrontation stress [64]. The increase in SERT in the limbic structures of the brain is frequently found after stressors of various types [65]. In contrast, a decrease in SERT expression in similar structures was shown to be a molecular correlate of clinical depression [66] and of an experimentally induced depressive-like state in animals [67]. These data, in combination with our own, suggest changes in molecular signals within specific brain regions may result in behaviourally distinct outcomes.

In vitro and in vivo studies have shown that pro-inflammatory cytokines, such as IL-1β and TNFα, can increase SERT activity via the p38 MAPK signalling pathway [46]. Behavioural signs of helplessness resulting from circulating cytokines have been shown to be prevented by a blockade of SERT [68]. Furthermore, SERT mutant rats show abnormal behaviour (including decreased sucrose preference, decreased spontaneous activity and increased anxiety [69]) and CNS cytokine expression profiles in response to LPS [70]. In humans, however, the reverse appears to be true. Clinical studies reveal that decreased SERT function, associated with the short variant of the SERT gene and lower SERT activity, correlates with an increased risk of developing depression during IFN-α treatment [71]. Indeed, our own work has demonstrated that there is no change in the release of 5-HT in response to LPS, suggesting a post-synaptic mechanism may be more crucial to sickness behaviour [72]. Thus, the relationship between SERT activity and responsiveness to pro-inflammatory factors in the regulation of depression pathogenesis appears to be complex and is liable to explain the differences we observed in aggressive behaviour associated with stress alone vs stress in combination with an inflammatory challenge.

The levels of 5-HT_2A_ mRNA were different in mice subjected to stress alone to those additionally challenged with LPS. Previously, elevation of 5-HT_2A_ in the limbic structures was documented as an important correlate of a depressive-like state, which represents a target for pharmacological treatment [73]. In line with our previous observations [29, 72], such changes were found in the pre-frontal cortex of stressed mice but not in naïve or stressed mice injected with LPS. However, a significant elevation of 5-HT_2A_ expression was detected in the hippocampus of the two latter groups, in line with similar findings elsewhere showing that inflammation significantly affects 5-HT_2A_ [35, 74]. The similarities in receptor expression profiles regardless of stress exposure suggest that changes in the expression of the 5-HT_2A_ receptor are unlikely to mediate the exacerbated behavioural effects observed in the double-hit mice.

Importantly, our low-dose LPS challenge in naïve animals resulted in the over-expression of TNFα in several brain structures, including the pre-frontal cortex, but this was not associated with alteration in the behaviours tested. Such findings are in accord with previously published results, showing that cytokine over-expression exerts minimal effects on social behaviour in rodents [56]. The expression of IL-1β in the dorsal raphe nucleus was significantly elevated in both naïve and stressed LPS-treated groups. However, this effect is also unlikely to underlie behavioural differences between chronically stressed mice, with or without LPS challenge, since naïve mice showed no obvious behavioural changes in aggression or depressive-like behaviours.

Stress is well known to increase circulating cortisol, and there is evidence linking cortisol levels and depression. Depressed patients frequently show dexamethasone non-suppression, suggesting hyperactivity of the Hypothalamic–pituitary–adrenal HPA axis [75]. Corticosterone levels are similar in animals subjected to either CMS or LPS and thus could not explain the phenotypic differences observed between stressed and LPS-challenged animals. These data are in line with previously reported findings [76] although oddly, the increase in corticosterone as a result of stress does not appear to reduce the hepatic inflammatory response. This data, and that in adrenalectomized animals, suggests that the pro-inflammatory profile during stress is independent of cortisol and may be the result of anti-inflammatory cytokines and downstream signalling pathways [77].

## Conclusions

Here, we have shown that the effects of chronic stress and LPS are reflected by dissociated alterations in both behaviour and gene expression, with elevated SERT expression appearing to be linked to stress-induced aggression. Furthermore, we have found that the molecular and behavioural changes induced by stress or low-grade inflammatory challenges are distinct and, when the challenges were combined, some of the behaviours synergized and others, such as the aggressive behaviours, were suppressed. It seems likely that distinct mechanisms enabling the body to effectively deal separately with stress vs infection have evolved but there is no doubt that the presence of low-grade inflammation can have a profound effect on stress-induced behaviours; the underlying mechanisms are likely to be of relevance in humans, where such combinations may precipitate depressive episodes.

## Additional file

Additional file 1: Table S1. Primer sequences for qPCR. Primers were custom designed and validated by PrimerDesign Ltd. (Southampton, UK). **Figure S1.** The effect of a low dose of LPS on locomotor activity at 24 and 48 h post-challenge in naïve mice. Naïve animals were subjected to a single dose of LPS (0.1 or 0.5 mg/kg) or vehicle injection and were tested at 24 or 48 h post-injection. (A) Neither the resting time was unaltered by the treatment in the TruScan open field nor (B) rearing in the novel cage test for the total number of rear. (C–E) Aggressive behaviour was also unaltered. Data are mean ± SEM, two-way ANOVA throughout. **Figure S2.** (A, B) Body weight in the chronic stress experiment. Experimental groups were balanced upon baseline mean values of body weight measured 7 days prior the start of the chronic stress experiment and LPS challenge. Mice exposed to chronic stress had a significant reduction in body weight as compared with baseline measurements (**p* < 0.05, pairwise *t* test). Chronically stressed mice injected either with vehicle or LPS had similar mean body weight prior the LPS challenge. (C–E) Sucrose preference. Experimental groups were balanced upon baseline mean values of sucrose preference when evaluated 7 days prior the experiment chronic stress procedure and LPS challenge. Experimental groups had similar mean measures of sucrose and water intake. (*p* > 0.05, one-way ANOVA and post hoc Tukey test; see the text). (F) Naïve and stressed animals (10 days) were challenged with a single dose of LPS (0.1 mg/kg) or vehicle (saline) and tested 24 h thereafter in a novel cage test for total number of rears (see the text). Data are mean ± SEM. No differences between the groups were observed. Figure S3. (A–C) Baseline behaviour in a resident-intruder test. Experimental groups were balanced upon baseline mean scores of behaviours in a resident-intruder test that were studied 7 days prior the experimental chronic stress procedure and LPS challenge. Mice had similar mean measures of (A) latency to attack, (B) number of attack and (C )duration of crawl over behaviour. (*p* > 0.05, one-way ANOVA and post hoc Tukey test; see the text). (D) The latency to attack after the chronic stress was not significantly altered.
