# Supplementary material for: Low-dose lipopolysaccharide (LPS) inhibits aggressive and augments depressive behaviours in a chronic mild stress model in mice
Source: J Neuroinflammation. 2016 May 16;13:108. doi: 10.1186/s12974-016-0572-0 (PMC4867526; doi:10.1186/s12974-016-0572-0)
Supplement: Additional file 1: Table S1. — Primer sequences for qPCR. Primers were custom designed and validated by PrimerDesign Ltd. (Southampton, UK). Figure S1. The effect of a low dose of LPS on locomotor activity at 24 and 48 h post-challenge in naïve mice. Naïve animals were subjected to a single dose of LPS (0.1 or 0.5 mg/kg) or vehicle injection and were tested at 24 or 48 h post-injection. (A) Neither the resting time was unaltered by the treatment in the TruScan open field nor (B) rearing in the novel cage test for the total number of rear. (C–E) Aggressive behaviour was also unaltered. Data are mean ± SEM, two-way ANOVA throughout. Figure S2. (A, B) Body weight in the chronic stress experiment. Experimental groups were balanced upon baseline mean values of body weight measured 7 days prior the start of the chronic stress experiment and LPS challenge. Mice exposed to chronic stress had a significant reduction in body weight as compared with baseline measurements (*p < 0.05, pairwise t test). Chronically stressed mice injected either with vehicle or LPS had similar mean body weight prior the LPS challenge. (C–E) Sucrose preference. Experimental groups were balanced upon baseline mean values of sucrose preference when evaluated 7 days prior the experiment chronic stress procedure and LPS challenge. Experimental groups had similar mean measures of sucrose and water intake. (p > 0.05, one-way ANOVA and post hoc Tukey test; see the text). (F) Naïve and stressed animals (10 days) were challenged with a single dose of LPS (0.1 mg/kg) or vehicle (saline) and tested 24 h thereafter in a novel cage test for total number of rears (see the text). Data are mean ± SEM. No differences between the groups were observed. Figure S3. (A–C) Baseline behaviour in a resident-intruder test. Experimental groups were balanced upon baseline mean scores of behaviours in a resident-intruder test that were studied 7 days prior the experimental chronic stress procedure and LPS challenge. Mice had similar mean measures of (A) lat [file 12974_2016_572_MOESM1_ESM.pdf]

Supplementary table (ST1): Primer sequences for qPCR. Primers were custom designed and validated by Primer Design Ltd. (Southampton, UK)

Marker	Forward primer	Reverse primer	Amplicon size (nt)
Tnf	GCTCCCTCTCATCAGTTCTAT	TTTGCTACGADCTGGGCTA	94
IL-1β	CAACCAACAAGTGTATTCTCCAT	GTGTGCCGTCTTTCATTA	127
5-HT$_{2A}$	CAGGCAAGTCACAGGATAGC	TTAAGCAGAAAGAAAATCCCACAG	93
Sert	TGCCTTTTATATCGCCTCCTAC	CAGTTGCCAGTGTTCCAAGA	127

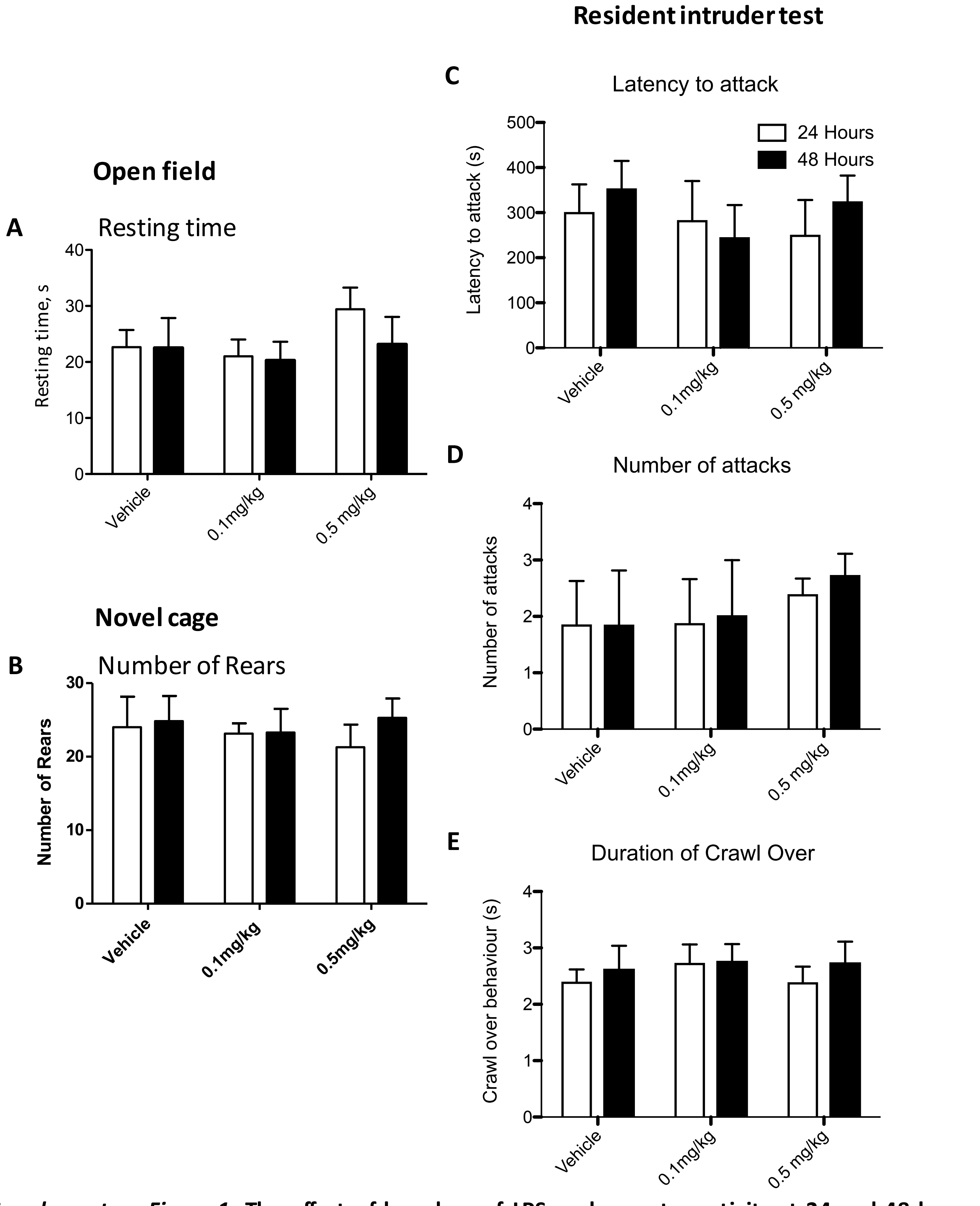

Supplementary Figure 1. **The effect of low dose of LPS on locomotor activity at 24 and 48 hours post-challenge in naïve mice.** Naïve animals were subjected to a single dose of LPS (0.1 mg/kg or 0.5 mg/kg) or vehicle injection, and were tested at 24 h or 48 h post-injection. **(A)** Neither the resting time was unaltered by the treatment in the Truescan open field nor **(B)** rearing in the novel cage test for total number of rear. **(C-E)** Aggressive behaviour was also unaltered. Data are mean ±SEM, 2-way ANOVA throughout.

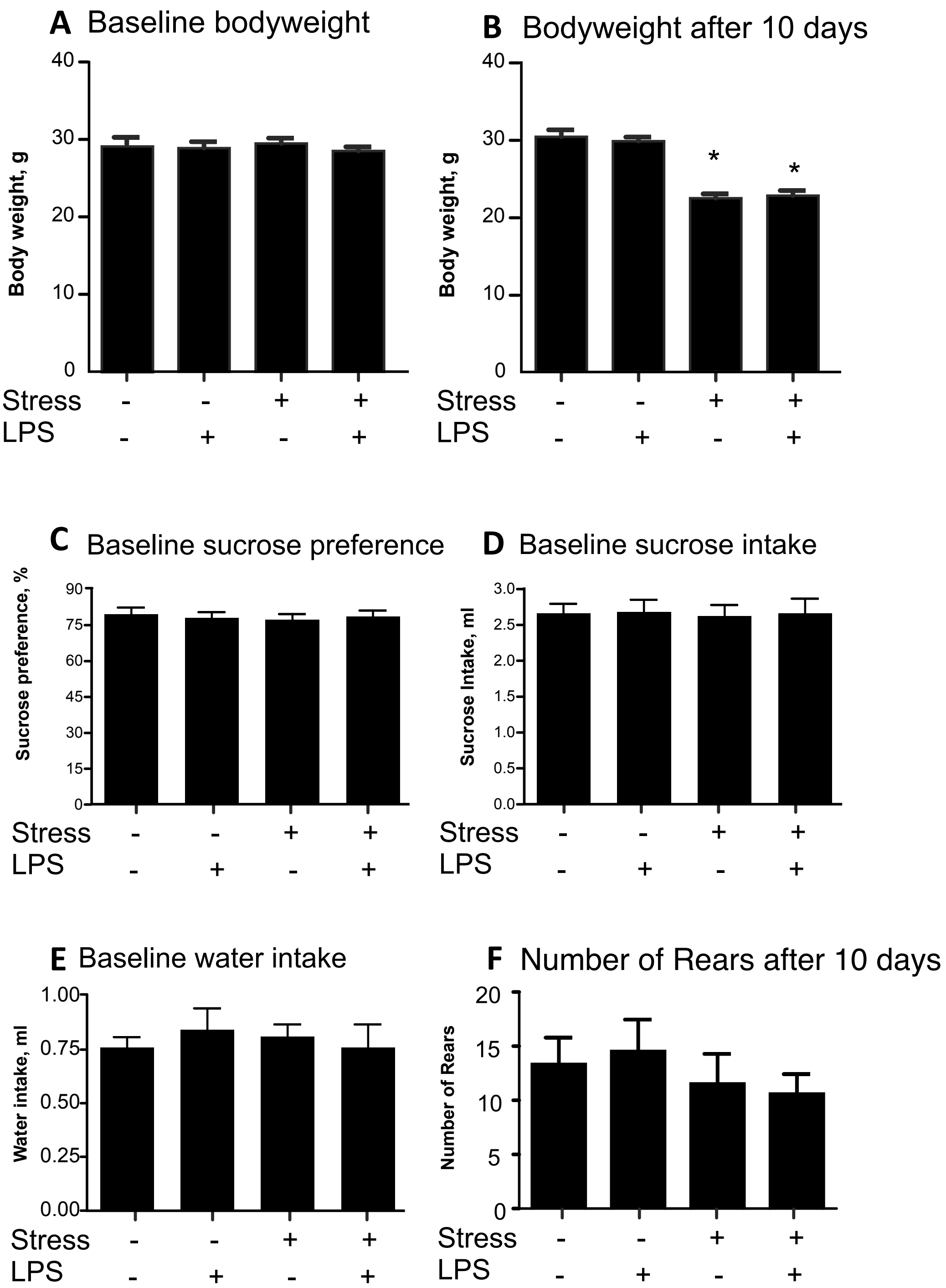

Supplementary Figure 2. (A&B) Body weight in the chronic stress experiment. Experimental groups were balanced upon baseline mean values of body weight measured seven days prior the start of the chronic stress experiment and LPS challenge. Mice exposed to chronic stress had a significant reduction in body weight as compared with baseline measurements (p*<0.05, pair-wise t-test). Chronically stressed mice injected either with vehicle or LPS had similar mean body weight prior LPS challenge. ***(C,D&E) Sucrose preference.*** Experimental groups were balanced upon baseline mean values of sucrose preference when evaluated seven days prior the experiment chronic stress procedure and LPS challenge. Experimental groups had similar mean measures of sucrose and water intake. ($p>0.05$, one-way ANOVA and post-hoc Tukey test; see the text). **(F)** Naïve and stressed animals (10 days) were challenged with a single dose of LPS (0.1 mg/kg) or vehicle (saline) and tested 24 thereafter in a novel cage test for total number of rears (see the text). Data are mean ±SEM. No differences between the groups was observed.

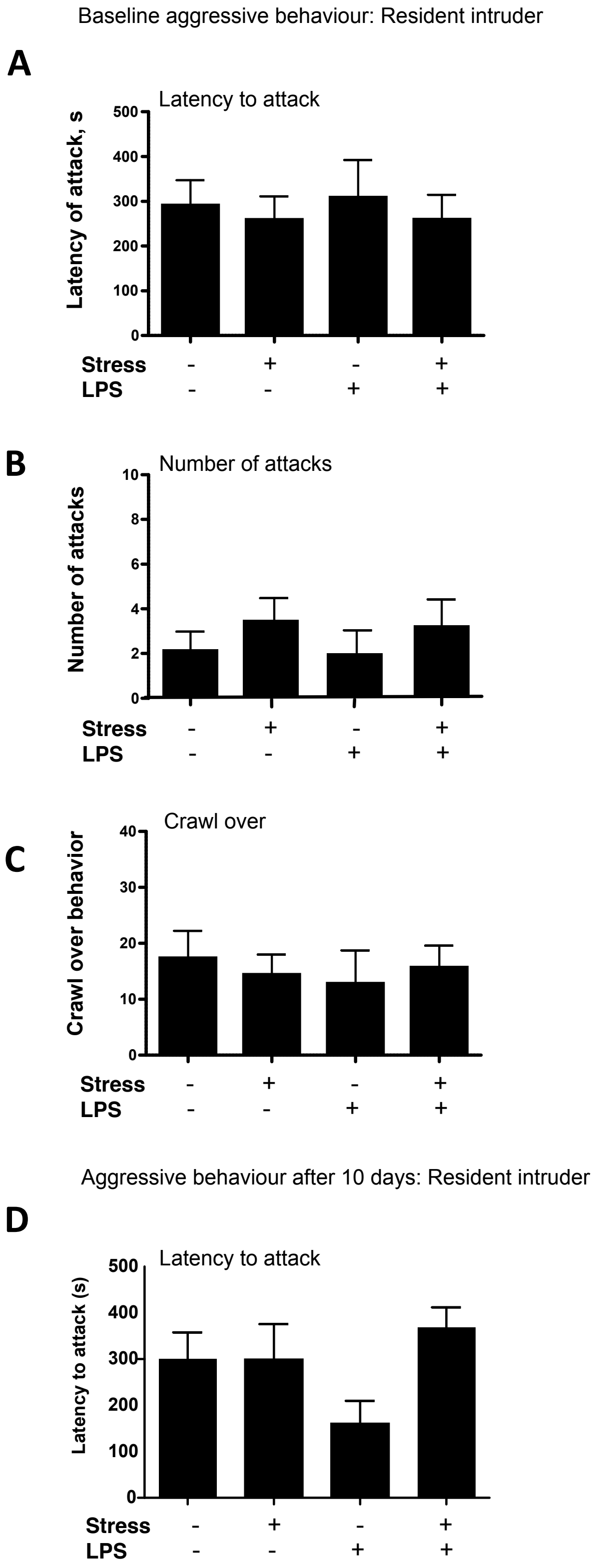

Supplementary Figure 3. (A, B, &C) Baseline behaviour in a resident-intruder test. Experimental groups were balanced upon baseline mean scores of behaviours in a resident-intruder test that were studied seven days prior the experimental chronic stress procedure and LPS challenge. Mice had similar mean measures of **(A)** latency to attack, **(B)** number of attack and duration of crawl over behaviour **(C)**. ($p>0.05$, one-way ANOVA and post-hoc Tukey test; see the text). **(D)** The latency to attack after the chronic stress was not significantly altered.
